# Enhancement of Fe–N–C carbon catalyst activity for the oxygen reduction reaction: effective increment of active sites by a short and repeated heating process†

**DOI:** 10.1039/c8ra08359b

**Published:** 2018-11-08

**Authors:** Satoshi Yasuda, Yosuke Uchibori, Makoto Wakeshima, Yukio Hinatsu, Hiroaki Ogawa, Masahiro Yano, Hidehito Asaoka

**Affiliations:** Research Group for Nanoscale Structure and Function of Advanced Materials, Advanced Science Research Center, Japan Atomic Energy Agency 2-4 Shirakata, Tokai Ibaraki 319-1195 Japan yasuda.satoshi@jaea.go.jp; Department of Chemistry, Faculty of Science, Hokkaido University Sapporo Hokkaido 060-0810 Japan; Research Group for Radiation Materials Engineering, Nuclear Science and Engineering Center, Japan Atomic Energy Agency 2-4 Shirakata Ibaraki 319-1195 Japan

## Abstract

Controlling the formation of Fe–N–C catalytic sites is crucial to activate the oxygen reduction reaction (ORR) for realization of non-precious electrocatalysts in proton exchange membrane fuel cells (PEMFCs). We present a quantitative study on the effect of a newly obtained thermal history on the formation of Fe–N–C catalytic sites. A short and repeated heating process is employed as the new thermal history, where short heating (1 min) followed by quenching is applied to a sample with arbitrary repetition. Through electrochemical quantitative analysis, it is found that the new process effectively increases the Fe–N–C mass-based site density (MSD) to almost twice that achieved using a conventional continuous heating process, while the turn-over frequency (TOF) is independent of the process. Elemental analysis shows that the new process effectively suppresses the thermal desorption of Fe and N atoms during the initial formation stage and consequently contributes to an increase in the Fe–N–C site density. The resultant catalytic activity (gravimetric kinetic current density (0.8 V *vs.* RHE)) is 1.8 times higher than that achieved with the continuous heating process. The results indicate that fine control of the thermal history can effectively increase the catalytic activity and provide guidelines for further activation of non-precious ORR electrocatalysts for PEMFCs.

## Abstract 2

Controlling the formation of Fe–N–C catalytic sites is crucial to activate the oxygen reduction reaction (ORR) for realization of non-precious electrocatalysts in proton exchange membrane fuel cells (PEMFCs).

## Graphical abstract

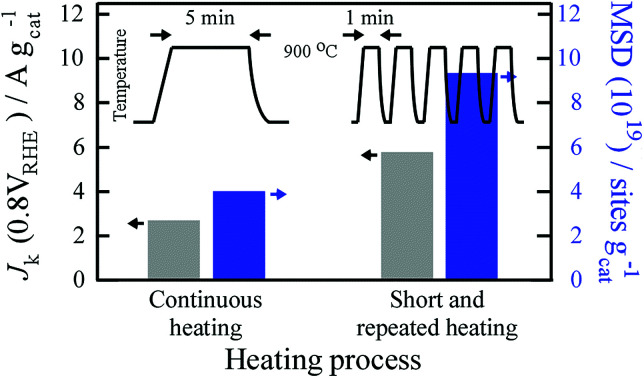

## Introduction

Proton exchange membrane fuel cells (PEMFCs) are energy conversion systems that directly convert the chemical energy of hydrogen and oxygen into electricity. They are promising candidates for clean and sustainable energy conversion. The oxygen reduction reaction (ORR) on the cathode in acid media is a key reaction in this conversion system, and Pt-based alloys supported by graphitic carbon are used as the electrocatalyst. However, the precious metal is expensive and its availability is limited. Therefore, the development of non-precious metal electrocatalysts for the ORR is highly desirable for facilitating worldwide use of PEMFCs.

Since the discovery of the ORR capability of non-precious metal electrocatalysts by Jasinski^1^ and Jahnke,^2^ the development of non-precious iron–nitrogen–carbon (Fe–N–C) catalysts has attracted considerable attention from the viewpoint of developing alternatives to precious Pt-based catalysts for ORR on cathodes because the catalysts exhibit high ORR performance in both acidic and alkaline media.^3–6^ Heating a mixture of Fe and N containing precursors and carbon-supporting materials effectively produces Fe–N–C catalytic sites on the carbon-supporting material, which is the iron (Fe)-coordinated nitrogen (N)-atom-functionalized graphitic carbon (C) structure. However, despite numerous studies, improvement of the ORR activity for realizing alternatives to Pt-based catalysts remains challenging.

Controlling the thermal history during Fe–N–C formation is of paramount importance for activation, and many researchers have studied the effect of heating conditions on Fe–N–C formation. For example, in an investigation of temperature and heating time dependence of ORR activity, it was found that prolonged continuous heating decreases ORR activity because it causes not only desorption of doped N atoms but also formation of Fe-based nanoparticles, thus decreasing Fe–N–C catalytic site density.^7^ From the heating temperature dependence, an optimum heating temperature at which ORR activity is maximized, thus influencing active site formation, was determined.^8,9^ However, there is a paucity of experimental studies on effect of thermal history on ORR activity. In this study, we focus on fine modulation of thermal history during Fe–N–C formation. The newly developed thermal history is a short and repeated heating process, and short heating (∼1 min) followed by quenching is applied to a sample with arbitrary repetitions. According to previous studies, Fe–N–C formation process is considered as follows: under heating, thermally decomposed Fe atoms in precursors diffuse and bind to N atoms functionalized at carbon micropore structures to form Fe–N–C catalytic sites. Simultaneously, nucleation and growth of Fe-based nanoparticles and thermal desorption of Fe and N atoms occur in the catalysts.^5,7,9^ The model strongly suggests that fine control of the diffusion and desorption of Fe and N during the formation process plays significant role in the formation of catalytic sites. Considering supposition, the new process would allow for control over the kinetics of thermal desorption and diffusion of Fe, N, and C in a catalyst during the formation process by changing the repetition number, thus facilitating fine control over Fe–N–C site formation and enhancing ORR performance. For comparison, we employed the commonly-used continuous heating process as well, in which a sample is heated continuously for a predetermined duration. To understand the difference in thermal history, gravimetric kinetic current density (*J*_k_), mass-based site density (MSD), and turn-over frequency (TOF), which are related directly to catalytic site performance, were elucidated by using the electrochemical redox method.^10^ We found that the short and repeated pyrolysis process suppresses thermal desorption of Fe and N atoms and consequently has a significant effect on the increase in MSD but no effect on TOF. The resulting catalysts exhibited *J*_k_ (0.8 V *vs.* reversible hydrogen electrode (RHE)) of *ca.* 7.9 A g^−1^ in an O_2_-saturated 0.5 M H_2_SO_4_, resulting in almost double the activity as that achieved using the conventional pyrolysis process.

## Experimental

### Catalyst preparation

**Fig. 1:**
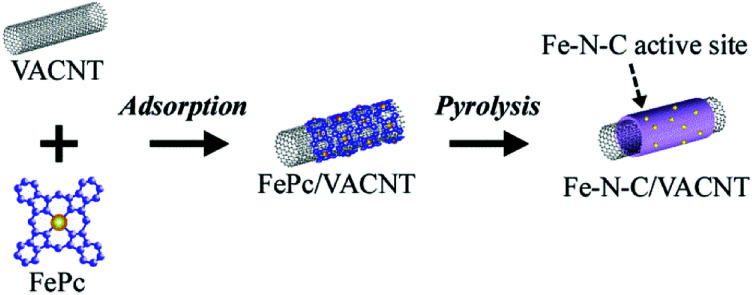
Adsorption of iron(ii) phthalocyanine (FePc) on VACNT to yield FePc/VACNT composite and subsequent heating to yield Fe–N–C/VACNT catalyst.

As carbon-supporting materials, vertically-aligned carbon nanotubes (VACNT) were used because they have a high specific surface area and highly aligned interstitial pores.^11,12^ The high specific surface area facilitates the immobilization of a large number of catalytic Fe–N–C sites on individual VACNT surfaces, realizing high ORR activity. In this study, VACNT was obtained from Zeon Co. Ltd. (product name of ZEONANOTMSG101), and the specific surface area of the VACNT was *ca.* 800 m^2^ g^−1^ as determined using N_2_ sorption measurement. The Fe–N–C catalysts were prepared by pyrolyzing a mixture of VACNT and iron(ii) phthalocyanine (FePc), as previously reported.^13^ Briefly, mixing of the VACNT and FePc produced FePc adsorbed VACNT composites owing to their strong affinity through π–π interaction, and the subsequent heating efficiently converted the FePc adsorbates into the Fe–N–C structures on the VACNT surface (Fig. 1). In this study, infrared lamp heating furnace was used because rapid heating and cooling can be carried out compared with a conventional resistance heating furnace, allowing the newly developed short and repeated heating process. Two different heating processes were employed to assess the influence of heating history on Fe–N–C formation, as shown in Fig. 3a and e. One is the conventional continuous heating process, in which composites are continuously heated to 700 °C min^−1^ and then maintained at 900 °C for an arbitrary duration. The other is short and repeated heating process, in which composites are heated for a short duration and the process is repeated arbitrarily. After heating at 700 °C min^−1^, the composites were maintained at 900 °C for 1 min, and then cooled at a rate of ∼300 °C min^−1^ for 2 min. The sequence was repeated for 1–10 cycles to control thermal history during Fe–N–C formation. In the process, heating time is expressed as “1 min × *n* heating cycle” to distinguish it from heating time in the continuous heating process. After heating, the obtained powders were breached by acidic solution to remove residual Fe particles produced by the heating, and then the powders were washed thoroughly with MilliQ water. The powders were heated again under a flow of diluted NH_3_/N_2_ gases, yielding the Fe–N–C/VACNT catalysts (see ESI†).

### Catalyst characterizations

Cyclic voltammetry (CV) and rotating disk electrode (RDE) measurements were conducted in O_2_- or Ar-saturated 0.5 M H_2_SO_4_ solution at room temperature. A RHE and Pt wire were used as the reference and the counter electrodes, respectively. All Fe–N–C/VACNT catalysts were loaded on a glassy carbon disk (0.6 mg cm^−2^) and used as the working electrode. RDE measurements were recorded a scan rate of 10 mV s^−1^ and a fixed rotation rate of 1600 rpm. The details of electrode preparation and electrochemical characterization are presented in the ESI.†

## Results and discussion

### Quantification of mass-based site density (MSD) and associated turn-over frequency (TOF) by electrochemical method

Intrinsic ORR catalytic activity of electrochemical reaction can be evaluated by gravimetric kinetic current density of ORR, expressed as follows:

1 *J*_k_ = *e* × TOF × MSD

where *J*_k_ (A g^−1^) is the gravimetric kinetic current density at a given potential; *e* is elementary charge; TOF (*e* site^−1^ s^−1^) is the turn-over frequency, and it is defined as the number of reacted electrons per active site per second; and MSD (sites g^−1^) is mass-based site density, and it is defined as the number of Fe–N–C site per unit mass of catalyst. Because TOF and MSD are correlated directly with catalytic site performance, quantitative evaluation of TOF and MSD provides further insight into the correlation between ORR activity and thermal history. Quantification of MSD and the associated TOF were carried out using the electrochemical redox method.^10^ Unlike previous studies, in which other methods such as XPS, ^57^Fe Mossbauer spectroscopy,^14,15^ gas desorption/adsorption,^16^ electrochemical molecular probe^17^ were used, the electrochemical redox method facilitates direct quantification of Fe–N–C sites on the surface of the catalysts and the associated TOF without any specific equipment and molecular probe.

**Fig. 2:**
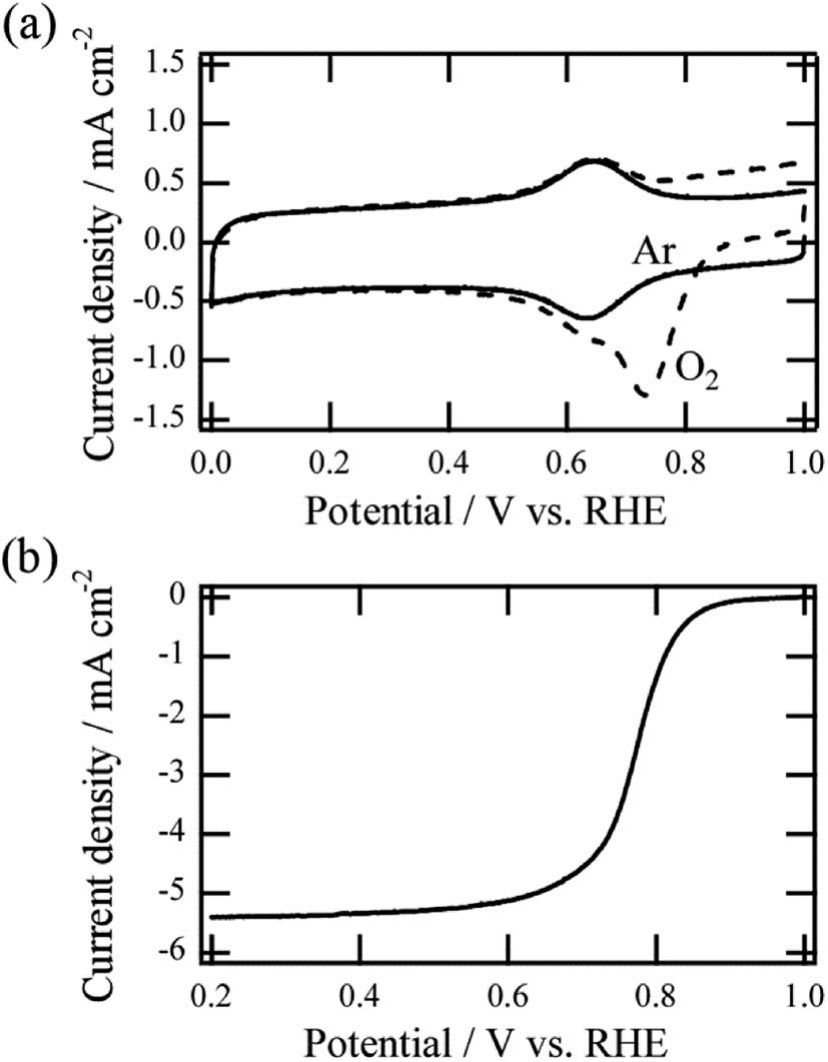
(a) Representative CV curves of Fe–N–C/VACNT catalysts in O_2_-saturated (dotted line) and Ar-saturated (solid line) 0.5 M H_2_SO_4_ solutions. (b) RDE curves of Fe–N–C/VACNT catalyst at 1600 rpm in the O_2_ saturated acid media.

Fig. 2a shows an example of cyclic voltammetry (CV) in Ar or O_2_-saturated acidic media for Fe–N–C/VACNT catalyst prepared by continuous heating (5 min continuous heating time). In the case of O_2_-saturated media, a larger cathodic peak at 0.73 V *vs.* RHE was observed, which was attributed to the ORR (dotted line).^13,18^ By contrast, reduction and oxidation (redox) peaks were observed around 0.65 V *vs.* RHE in Ar-saturated media (solid line). The peak currents as a function of scan rate (Fig. S1†) showed that both the anodic and the cathodic peak currents are directly proportional to the scan rate, indicating the surface-immobilized redox process. Previous studies showed that the redox peaks observed in Ar-saturated media can be attributed to accessible surface-based Fe–N–C sites.^18,19^*In situ* electrochemical X-ray adsorption spectroscopy revealed experimentally that the redox reaction can be ascribed to the Fe(ii)/Fe(iii) redox transition of the Fe–N–C site in the catalyst, and it is a reversible one-electron process.^18^

The MSD can be quantified by analyzing the redox peak area considering the one-electron process and the loaded weight of the catalyst. After CV measurements, the ORR polarization curve at 1600 rpm in O_2_-saturated acid media were evaluated (Fig. 2b). The curve can be used to calculate gravimetric kinetic current density *J*_k_ at 0.8 V *vs.* RHE, and the associated TOF can be evaluated using the estimated MSD value. All TOF values of catalysts were estimated at the potential of 0.8 V *vs.* RHE. Details of the estimation method are given in the ESI.† It is noted that Pt of the counter electrode was found to dissolve in the acidic medium and re-deposit on a non-precious metal catalyst on the working electrode during potential cycles, causing the ORR performance to improve.^20,21^ In this study, we confirmed that there is no such undesirable effect for all ORR measurements (Fig. S2†), and obtained all data of the catalysts reflects their own catalytic properties.

### Effect of thermal history on *J*_k_, TOF, and MSD

**Fig. 3:**
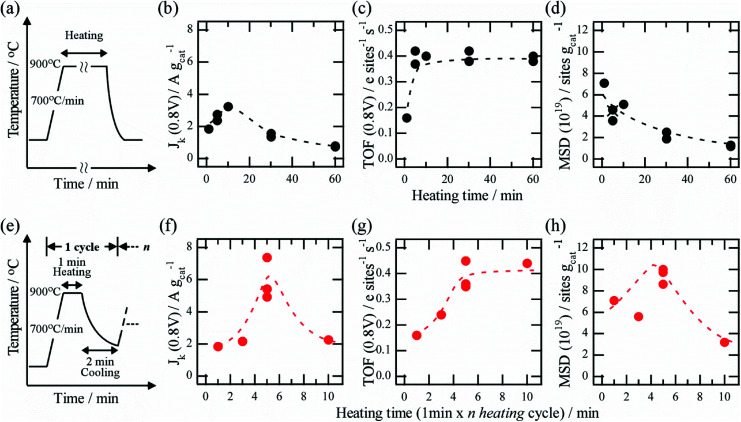
(a) Profile of continuous heating process. Time evolution of (b) *J*_k_, (c) TOF, and (d) MSD of catalyst prepared by a process. (e) Profile of short and repeated heating process. Time evolution of (f) *J*_k_, (g) TOF, and (h) MSD of catalyst prepared by (e) process.

Based on above the analysis, the effect of thermal history on the catalytic parameters was investigated quantitatively. Fig. 3a shows the temperature profile at which thermal history is applied continuously to a catalyst at 900 °C, and it is widely used as the heating process for catalyst preparation. The time evolution of *J*_k_ revealed that *J*_k_ peaks when the heating ranges from 5 to 10 min, and the ORR activity decrease for heating times shorter or longer than 5–10 min (Fig. 3b). *J*_k_ of the catalyst produced with a moderate heating time (5–10 min) was two or three times higher than that of the catalyst produced with a long heating time (60 min). The results indicate the importance of optimizing the heating time, which is consistent with the results of a previous study.^7^

To understand the observed trend of *J*_k_, time evolutions of the TOF and MSD were analyzed quantitatively. The time evolution of TOF showed an immediate increase within 5 min and termination over the subsequent 10 min with TOF of ∼0.4 (*e* site^−1^ s^−1^) (Fig. 3c). By contrast, the time evolution of MSD was different from that of TOF, and it decreased monotonically as heating time increased (Fig. 3d). These results can explain the observed trend of *J*_k_: short heating time results in the lowest ORR activity, which can be ascribed to the low TOF value, while moderate heating time (∼5 min) results in the highest activity because of high TOF and MSD values. Longer heating times cause the MSD to decrease significantly and the activity to decrease. Similar quantitation analysis was performed for the catalyst produced using the short and repeated heating process. Fig. 3e shows an illustration of the short and repeated heating process. It is noted again that *n* heating time in the process is expressed as “1 min × *n* heating cycle” (*n* is equal to cycle number) to distinguish this from the heating time in the continuous heating process. The time evolution of *J*_k_ for the process showed a similar peak trend to that of the continuous heating process. Interestingly, we found that the peak value at optimized condition was 1.8 times higher than that in the continuous heating process (Fig. 3b and f), showing significant enhancement of the catalytic activity without any additional precursors or gases during pyrolysis process. The result clearly indicates an importance of optimization of the thermal history. Quantitative analysis revealed that the trend of TOF was similar to that of the continuous heating process, and the value tended to saturate ∼0.4 at around 1 min × 5 heating cycles (Fig. 3g). By contrast, unlike the monotonically decreasing trend of MSD in case of the continuous heating process, the time evolution of MSD peaked at around 1 min × 5 heating cycles (Fig. 3h). Based on these quantitative analysis results, we found that for both processes, the values of TOF and MSD depend significantly on the heating time, and consequently, *J*_k_ affect the trends of MSD and TOF. A comparison of both results also indicates that the difference in thermal history significantly influences MSD, whereas TOF is almost independent of the thermal history. The values of MSD, TOF, and *J*_k_ when *J*_k_ peaks are listed in Table 1 for different heating processes. Interestingly, the MSD for the short and repeated process with 1 min × 5 heating cycles is 9.4 × 10^19^ (sites g^−1^), which is almost twice that in case of the continuous process with 10 min of heating time (5.1 × 10^19^ sites g^−1^). The enhancement of *J*_k_ in case of the short and repeated heating cycle can mainly be attributed to the increase in MSD because the TOFs of both processes are almost equal.

**Table 1:** Representative values of *J*_k_, TOF, and MSD for each process

Process	*J*_k_ (A g^−1^)	TOF (*e* site^−1^ s^−1^)	MSD (sites g^−1^)
Continuous heating (10 min)	3.2	0.40	5.1 x 1019
Short and repeated heating (5 min)	5.9	0.39	9.4 x 1019

**Fig. 4:**
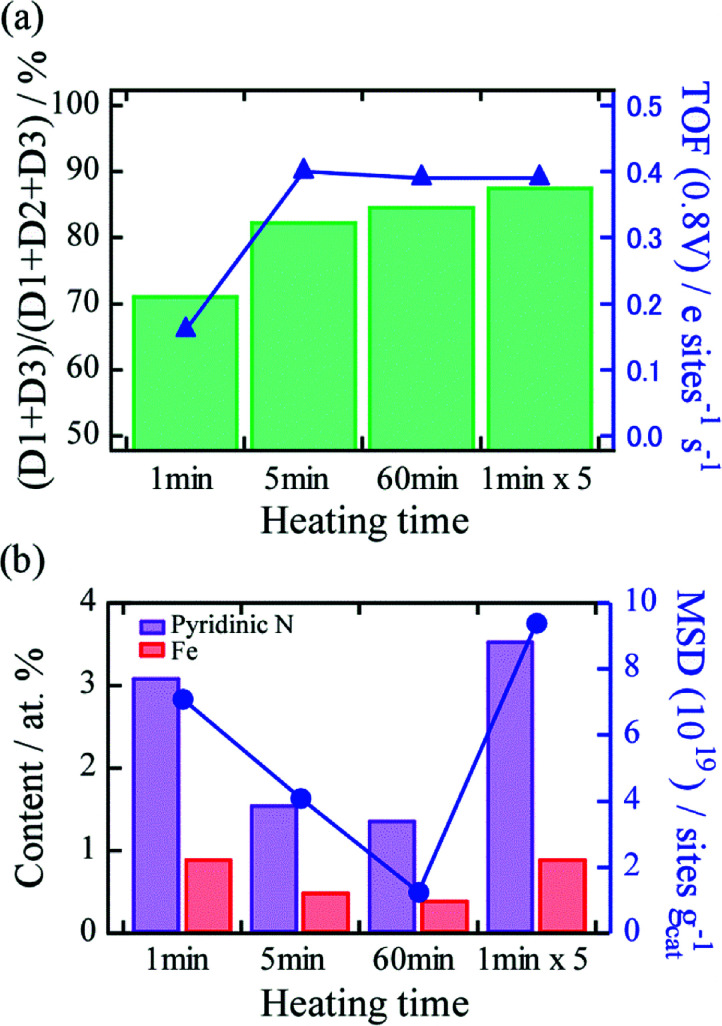
(a) Relationship between fraction of D1 + D3 active sites to D1 + D2 + D3 and TOF for each heating time. (b) Relationship between MSD and contents of pyridinic N and Fe produced by each heating time.

To obtain deep insights into the effect of differences in thermal history on TOF and MSD, ^57^Fe Mössbauer spectroscopy and XPS were conducted. First, Fe–N–C active sites in the catalysts were characterized using Mössbauer spectroscopy. The spectra of the catalysts prepared by 1, 5, and 60 min continuous heating, and by 1 min × 5 cycles of short and repeated heating were fitted with three doublets, two sextets, and a singlet (Fig. S2†), and the deconvolution results are summarized in Table S1.† The doublets D1, D2, and D3 correspond to the square-planar Fe(ii)–N_4_–C structure in the low-spin, intermediate, and high-spin states, respectively.^15^ It was reported that D1 and D3 correspond to major ORR active Fe–N–C structures because of the presence of unoccupied states of the 3d_*z*_^2^ orbital of Fe for O_2_ adsorption. The two sextets and singlets can be assigned to α-Fe, Fe_3_C, and superparamagnetic α-Fe or γ-Fe, respectively, and these peaks indicate the presence of Fe-based nanoparticles.^15,22,23^ We found that for the heating time of even 1 min, the Fe-based nanoparticles were produced in the catalyst, and the fraction of nanoparticles increased with heating time (Table S1†). The fraction of D1 and D3 active sites to all Fe–N–C sites ((D1 + D3)/(D1 + D2 + D3)) and the TOF values for the heating times were plotted, as shown Fig. 4a. We found that the fraction tended to increase with heating time (71%, 83%, 85%, and 88% at heating times of 1 min, 5 min, 60 min, and 1 min × 5 heating cycles, respectively), suggesting that the low TOF value at 1 min heating time could be associated with the low fraction value. Furthermore, no significant difference in the fractions was observed between the catalysts produced by 5 min continuous heating and 1 min × 5 heating cycles. This result implies that heating time plays an important role in the formation of the D1 and D3 active sites, and differences in thermal history, such as heating and quenching cycles hardly influence the formation. The effect of the difference in thermal history on MSD was investigated using XPS. Elemental analysis showed that the catalysts feature a prominent graphitic C 1s peak at ∼284 eV, N 1s peaks at ∼400 eV, an O 1s peak at ∼530 eV, and Fe peaks at ∼700 eV (Fig. S3†). The relative atomic concentrations of each element are listed in Table S2.† Two Fe 2p peaks at *ca.* 711 and 725 eV were observed, which correspond to the lower (Fe 2p_3/2_) and higher (Fe 2p_1/2_) energies due to spin–orbital splitting. An N 1s peak can be deconvoluted into three N bonding configurations, namely, pyridinic (398.7 eV), pyrrolic (400.3 eV), and quaternary (401.2 eV) (Table S3†).^24–26^ Pyridinic N is located at the edge of the graphene plane and is thus an indispensable component for Fe–N_4_ active site formation. As for the continuous heating process, we found that not only the relative concentrations of N and Fe but also the fraction of pyridinic N in the catalysts decreased with increasing heating time (Fig. 4b). These results strongly suggest that longer heating time causes thermal desorption of Fe and N atoms and decomposition of pyridinic N, leading to decreased MSD. By contrast, we found that the concentrations of Fe and N atoms, and pyridinic N in the catalyst produced by 1 min × 5 short and repeated heating cycles are similar to those produced by heating for 1 min, which is twice as high as that produced by continuous heating for 5 min, even though the heating time are similar. This result indicates that with the optimum number of repetitions of heating (5 cycles in this case), the short and repeated process effectively suppresses thermal desorption of N and Fe compared to the continuous heating process, thus contributing to an increase in MSD. Based on these analyses, we revealed that the differences in the thermal history in terms of repetitions of heating and quenching strongly affect MSD.

The effective increase in MSD due to the short and repeated heating process can possible be ascribed to the formation of the metastable states of Fe and N owing to the quenching effect according to the crystal growth model.^27^ With the initial heating, the FePc molecules adsorbed on VACNT surface decompose thermally, and individual Fe and N atoms disperse on the surface. Simultaneously, thermal diffusion and desorption of Fe and N atoms, formation and decomposition of Fe–N–C sites, and nucleation of Fe-based nanoparticles owing to the coalescence of diffusing Fe atoms occur.

In case of the continuous heating process, a longer heating time would not only increase the amounts of thermal desorption of Fe and N atoms in the catalyst but also lead to the formation of Fe-based nanoparticles owing to preferential Gibbs free energy change. Consequently, the number of the Fe–N–C site decreases, causing a monotonic decrease in MSD, as shown in Fig. 3d. Meanwhile, short and repeated pyrolysis process would take different feature. After the initial 1 min of heating, quenching would produce metastable compounds of Fe atoms bonded with carbons or nitrogen. For example, iron nitride compounds produce various phases, and theoretical calculation suggests that almost phases are metastable.^28^ The metastable compounds would serve as pinning and effectively suppress the thermal desorption of Fe and N atoms, thus contributing to an increase in MSD at the optimum number of heating repetitions. Increasing the number of repetitions beyond the optimum value would decrease MSD because Fe atoms from thermally decomposed Fe–N–C sites would nucleate the particles and be incorporated into them. However, detail elucidation of the metastable compounds is necessary for further discussion. It is next challenge to comprehend the heating procedures dependence of MSD by analyzing the change of catalyst in the step by step during the continuous or short heating treatment using microscopic or X-ray absorption fine structure methods.

## Conclusions

We developed a higher-ORR activation method for Fe–N–C catalysts by focusing on the thermal history during the Fe–N–C formation process. The newly developed thermal history is a short and repeated heating process, in which short heating (1 min) followed by quenching is applied to a sample with arbitrary repetition. We found that the process effectively enhances the catalytic activity which has 1.8 times higher than that with the conventional continuous heating process. Through quantitative analysis, it was found that the new process suppresses the thermal desorption of Fe and N atoms by means of the quenching effect during the initial Fe–N–C catalytic site process, and it doubles the MSD compared to that achieved with the conventional process, in which a sample is heated continuously for catalytic site formation. By contrast, TOF was found to be independent of the process. Our results strongly indicate that fine control over the thermal history effectively enhance the catalytic activity without any additional precursors or gases during pyrolysis process and would serve as one of facile methods for designing highly active Fe–N–C ORR electrocatalysts for PEMFCs.

## Conflicts of interest

There are no conflicts to declare.

## Supplementary Material

RA-008-C8RA08359B-s001
