# Supplementary material for: Enhancement of Fe–N–C carbon catalyst activity for the oxygen reduction reaction: effective increment of active sites by a short and repeated heating process
Source: RSC Adv. 2018 Nov 8;8(66):37600–5. doi: 10.1039/c8ra08359b (PMC9089374; doi:10.1039/c8ra08359b)
Supplement: RA-008-C8RA08359B-s001 [file RA-008-C8RA08359B-s001.pdf]

Electronic Supplementary Material (ESI) for RSC Advances.
This journal is © The Royal Society of Chemistry 2018

Electronic Supplementary Information

Enhancement of Fe-N-C Carbon Catalysts Activity for Oxygen Reduction Reaction: Effecttive Increment of Active Site by Short and Repeated Heating Process

Satoshi Yasuda,[1] Yosuke Uchibori,[2] Makoto Wakeshima,[2] Yukio Hinatsu,[2] Hiroaki Ogawa,[3] Masahiro Yano,[1] and Hidehito Asaoka[1]*

[1] Research Group for Nanoscale Structure and Function of Advanced Materials, Advanced Science Research Center, Japan Atomic Energy Agency, 2-4 Shirakata, Tokai, Ibaraki, 319-1195, Japan

[2]Department of Chemistry, Faculty of Science, Hokkaido University, Sapporo, Hokkaido, 060-0810, Japan

[3]Research Group for Radiation Materials Engineering, Nuclear Science and Engineering Center, Japan Atomic Energy Agency, 2-4 Shirakata, Ibaraki 319-1195, Japan

Corresponding author : yasuda.satoshi@jaea.go.jp

Catalyst preparation

VACNT (4.0 mg) dispersed in 5mL of dichloromethane was homogenized in an ice bath, and then 1.6 mL of 10 mM FePc dispersed in CH_2Cl_2 was mixed with it. The FePc-adsorbed VACNT dispersed solution was dried under vacuum, yielding the FePc-adsorbed composites. The composites were heated to thermally transform the FePc to Fe-N-C catalytic sites on the VACNT surface, and consequently, the Fe-N-C/VACNT catalysts were produced. In this study, two different heating processes were employed, as described in the manuscript. During each heating process, the composites were heated under the flow of Ar (200 sccm) and diluted 1% NH_3/N_2 (150 sccm) gases. After heating, the obtained powders were washed with 0.5 M H_2SO_4 at 80 °C for 15 h to remove residual Fe particles produced by the heating, and then thoroughly washed with MilliQ water. The powders were heated again under flows of Ar and diluted NH_3/N_2 gases to obtain the Fe-N-C/VACNT catalysts.

Electrode preparation and electrochemical Characterization

Cyclic voltammetry (CV) and rotating disk electrode (RDE) measurements were conducted using RRDE-3A apparaturs (ALS Company, Japan). A reversible hydrogen electrode and Pt wire were used as the reference and counter electrodes, respectively. A glassy carbon disk (4 mm diameter) was used as the rotating working disk electrode. Catalyst ink was prepared by homoginating 2 mg of catalyst in 40 µL of ethanol, 160 µL of MilliQ water, and 1 µL of 5 wt.% Nafion solution for 3 min. 8 µL of ink was deposited on the glassy carbon disk and then dried at 60 °C for 1 h, resulting in a catalyst loading of 0.6 mg cm^{-2}.

CV measurements were conducted in O_2- or Ar-saturated 0.5 M H_2SO_4 solution at a scan rate of 10 mV/s. Linear sweep voltammograms in the RDE test were obtained in O_2-saturated 0.5 M H_2SO_4 solution. Before each measurement, the solution was bubbled with O_2 gas for 30 min, and the potential of the working electrode was cycled several times from 0.0 to 1.0 V at 50 mV s^{-1} until similar I–V shapes were observed. Electrochemical data were recorded from 0.0 to 1.0 V, with a scan rate of 10 mV s^{-1} and a fixed rotating rate of 1600 rpm. Corrected oxygen reduction currents were obtained by subtracting the background current obtained for the Ar-saturated solution from the measured current obtained for the O_2-saturated solution.

The mass-based site density (MSD) was elcuideted by electrochemical redox method[1] and calculated as follows:

$$\mathrm{MSD} = \frac{Q}{eng_{cat}}$$

where MSD is the number of Fe-N-C sites normalized by mass of the catalyst (sites g^{-1}), Q is $Fe^{2+/3+}$ columbic charge (C) calculated by the CV in Ar-saturated solution, e is elementary charge, n is reaction nubmer (n = 1 in this redox system), and g_{cat} is loaded weight of the Fe-N-C/VACNT catalyst.

Turnover frequency (TOF) was calculated using the following equation:

$$\mathrm{TOF} = \frac{J_{kin}(0.8V\ vs.RHE)}{MSD \times e}$$

where J_k (0.8 *V vs. RHE*) is the gravimetric kinetic current density (A g^{-1}) at 0.8V vs. RHE, and it is expressed as follows:

$$J_{kin}(0.8V\ vs.RHE) = \frac{J_{lim} \times J_{0.8V}}{J_{lim} - J_{0.8V}}$$

TOF is defined as the number of reacted electrons per active site per second (e $site^{-1}$ s^{-1}), J_{lim} is the gravimetric limiting current density, and $J_{0.8V}$ is the gravimetric current density observed at 0.8V vs. RHE. J_k, J_{lim}, and $J_{0.8V}$ can be evaluated from the linear sweep voltammograms in the RDE.

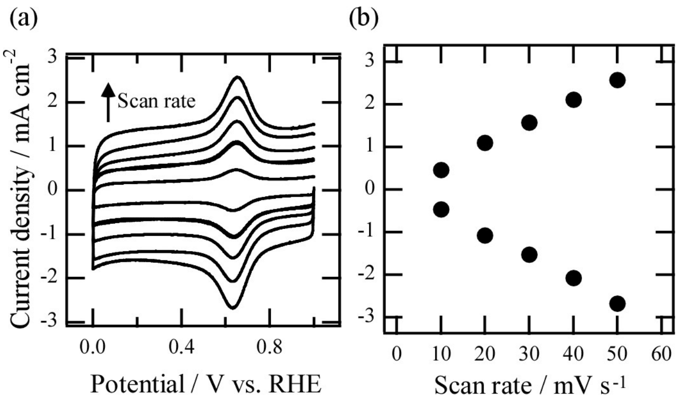

Figure S1. (a) Scan rate dependence of CVs and (b) plot of oxidation and reduction peak current densities versus scan rate for Fe-N-C/VACNT.

In this study, Pt wire was used as the counter electrode. It is reported that Pt of couner electrode is found to dissolve in acidic mediun and re-deposit on a non-precious metal catalyst coated on the working electrode during a repeated potential cycles.[2,3] Consequent re-deposition causes ORR performance of the catalyst to improve. For example, RDE curve of 1500 potential cylces of the non-precious metal catalyst from 0.6 to 1.2V (vs. RHE) in O_2-sturated acidic media showed 86 mV positively shift of half-wave potential, which is 67 mV higher than the initial one.[2]

In our study, all RDE curves of the Fe-N-C/VACNT catalysts were acquired after a few potential cycles from 0.0 to 1.0 V (vs. RHE) for a pretreatment, and the potential cycle number was much lower than the number that Platinum dissolves and re-deposites on the catalyst. Therefore, it is strongly suggested that no Pt re-deposition on the Fe-N-C/VACNT catalyst would occur in our experiment due to the extremely low cycle number.

To confirm that there is no Platinum re-deposition effect on the catalyst, the RDE curves after 1st and 50th potential cycles were compared. As shown in **Figure S2,** no significant change of ORR curves and half-wave potentials (both values :0.73V) were observed even though 50th potential cycles. The results are clear evidence that obtained all data of the catalysts reflects their own catalytic properties.

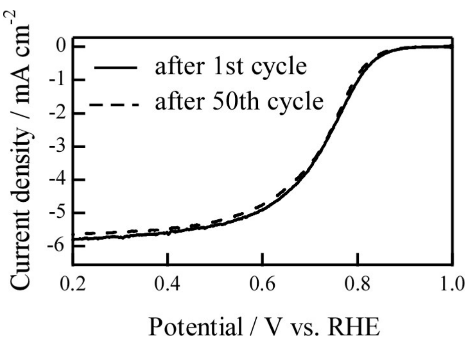

Figure S2. RDE curves of Fe-N-C/VACNT catalyst at 1600 rpm in the O_2-saturated 0.5 M H_2SO_4 solutions after 1st and 50th potential cycles. Potential range was from 0.2 to 1.0V at 50mV/s.

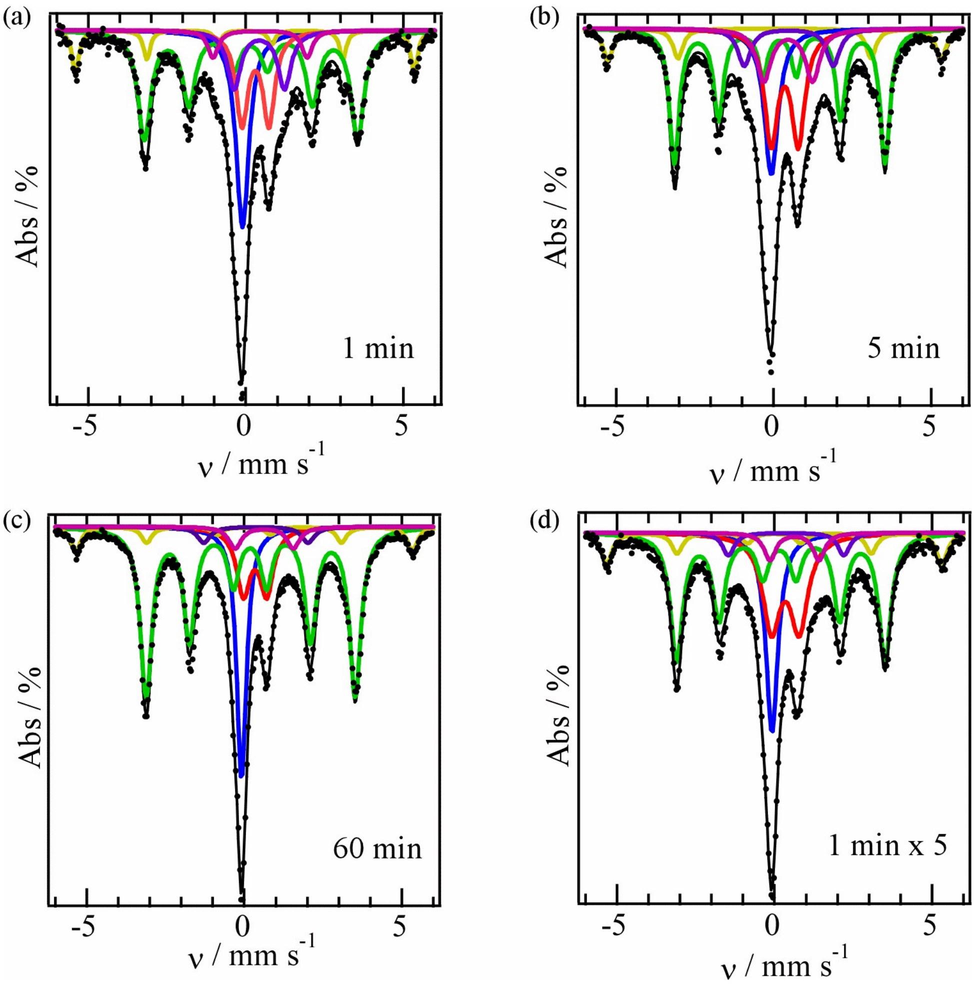

Figure S3. ^{57}Fe Mössbauer spectra of catalysts prepared by (a) 1 min, (b) 5 min, (c) 60 min, and (d) 1 min × 5 heatimg cycles.

Components	Relative ratio / %			
	1 min	5 min	60 min	1 min x 5
Singlet	19.8	16.3	17.6	16.9
Sextet 1	9.8	12.0	6.9	9.3
Sextet 2	37.7	37.6	55.8	42.9
Fe compounds total	67.3	65.9	80.3	69.1
Doublet D1	18.4	18.1	12.7	22.6
Doublet D2	9.4	6.0	3.0	3.8
Doublet D3	4.9	10.0	4.0	4.5
Doublet total	32.7	34.1	19.7	30.9

Table S1. Assgiement and amounts of calculated peak deconvolutions of Mössbauer spectrum.

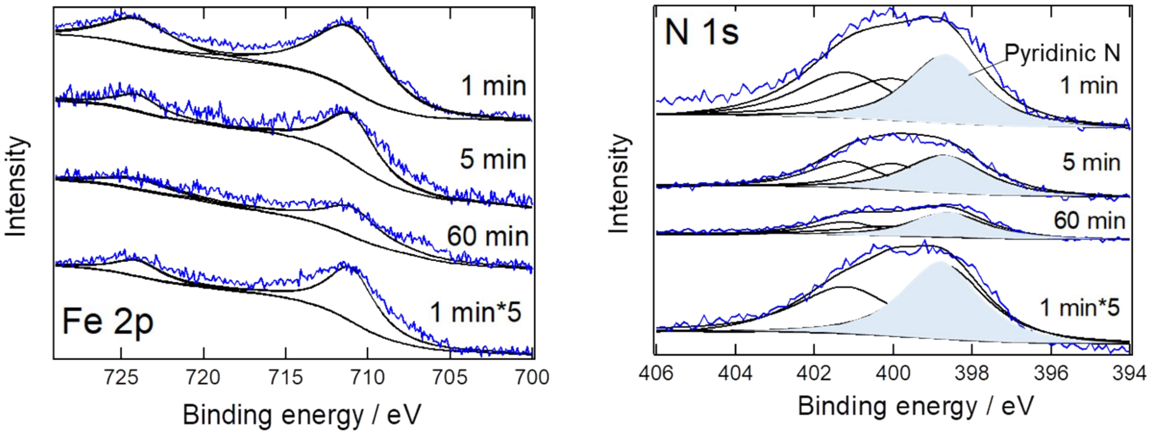

Figure S4. XPS spectra of Fe2p and N1s for catalysts prepared by 1 min, 5 min, 60 min, and 1 min × 5 min heating cycles.

Relative ratio (at. %)	Relative ratio / at %			
	1 min	5 min	60 min	1 min x 5
C	85.3	90.0	91.3	84.2
Fe	0.9	0.5	0.4	0.9
N	6.2	4.0	4.1	7.8
O	7.6	5.5	4.2	7.1

Table S2. Relative concentrations (atomic %) of C, Fe, N, and O atoms in catalysts prepared by 1 min, 5 min, 60 min, and 1 min × 5 heating cycles estimated by XPS.

N type (at. %)	Relative ratio / at %			
	1 min	5 min	60 min	1 min x 5
Pyridinic	49.9	39.0	33.5	45.4
Pyrrolic	24.1	19.8	26.5	20.8
Quaternary	26.0	41.2	40.0	33.8

Table S3. Relative contentrations (atomic %) of nitrogen species in catalysts prepared by 1 min, 5 min, 60 min, and 1 min × 5 heating cycles estimated by XPS.

References

(1) Ramaswamy, N.; Tylus, U.; Jia, Q.; Mukerjee, S. Activity Descriptor Identification for Oxygen Reduction on Nonprecious Electrocatalysts: Linking Surface Science to Coordination Chemistry. *J. Am. Chem. Soc.* **2013**, *135*, 15443–15449.

(2) Li, J.; Liu, H.; Lü, Y.; Guo, X.; Song, Y. Influence of Counter Electrode Material during Accelerated Durability Test of Non-Precious Metal Electrocatalysts in Acidic Medium. *Chinese J. Catal.* **2016**, *37*, 1109–1118.

(3) Chen, R.; Yang, C.; Cai, W.; Wang, H.-Y.; Miao, J.; Zhang, L.; Chen, S.; Liu, B. Use of Platinum as the Counter Electrode to Study the Activity of Nonprecious Metal Catalysts for the Hydrogen Evolution Reaction. *ACS Energy Lett.* **2017**, *2*, 1070–1075.
